# Removal of Emerging Contaminants and Estrogenic Activity from Wastewater Treatment Plant Effluent with UV/Chlorine and UV/H_2_O_2_ Advanced Oxidation Treatment at Pilot Scale

**DOI:** 10.3390/ijerph15050935

**Published:** 2018-05-07

**Authors:** Eduard Rott, Bertram Kuch, Claudia Lange, Philipp Richter, Amélie Kugele, Ralf Minke

**Affiliations:** Institute for Sanitary Engineering, Water Quality and Solid Waste Management, University of Stuttgart, Bandtäle 2, 70569 Stuttgart, Germany; bertram.kuch@iswa.uni-stuttgart.de (B.K.); claudia.lange@dekra.com (C.L.); philipp.richter@iswa.uni-stuttgart.de (P.R.); ask.kugele@t-online.de (A.K.); ralf.minke@iswa.uni-stuttgart.de (R.M.)

**Keywords:** AOP, AOX, emerging contaminants, estrogenic activity, UV/chlorine, UV/H_2_O_2_

## Abstract

Effluent of a municipal wastewater treatment plant (WWTP) was treated on-site with the UV/chlorine (UV/HOCl) advanced oxidation process (AOP) using a pilot plant equipped with a medium pressure UV lamp with an adjustable performance of up to 1 kW. Results obtained from parallel experiments with the same pilot plant, where the state of the art UV/H_2_O_2_ AOP was applied, were compared regarding the removal of emerging contaminants (EC) and the formation of adsorbable organohalogens (AOX). Furthermore, the total estrogenic activity was measured in samples treated with the UV/chlorine AOP. At an energy consumption of 0.4 kWh/m^3^ (0.4 kW, 1 m^3^/h) and in a range of oxidant concentrations from 1 to 6 mg/L, the UV/chlorine AOP had a significantly higher EC removal yield than the UV/H_2_O_2_ AOP. With free available chlorine concentrations (FAC) in the UV chamber influent of at least 5 mg/L (11 mg/L of dosed Cl_2_), the total estrogenic activity could be reduced by at least 97%. To achieve a certain concentration of FAC in the UV chamber influent, double to triple the amount of dosed Cl_2_ was needed, resulting in AOX concentrations of up to 520 µg/L.

## 1. Introduction

Anthropogenic compounds have been detected in wastewater treatment effluent, surface water, and ground water over the last years [1,2,3,4,5,6]. Many compounds, referred to as emerging contaminants (ECs), are brought into the environment by the disposed effluent of municipal wastewater treatment plants (WWTP) due to their stability against biological decomposition. These compounds may endanger aquatic life forms and, ultimately, humans via the food chain. Some endocrine disrupting compounds (EDCs) interfere with the hormone system [7]. After exposure, some compounds may cause cancer in humans [8]. Therefore, an obligatory, additional treatment step in WWTPs will be required.

Today, numerous alternative treatment methods for the removal of these compounds have been considered, including activated carbon treatment [9,10] or membrane filtration [11,12]. The advanced oxidation process (AOP) is a modern solution for the reduction of EC concentrations in wastewater treatment plant effluent (WWTE) [13]. In UV/AOP, an oxidant is dosed to the WWTE and activated by UV radiation to form highly reactive and unselective hydroxyl radicals (•OH). Hence, organic pollutants can be oxidized to CO_2_ and H_2_O or at least rendered biodegradable for subsequent natural degradation. State of the art oxidants are hydrogen peroxide (H_2_O_2_) and ozone (O_3_) [14,15].

UV/chlorine AOP is a promising alternative. In previous studies [16,17,18,19,20], good removal rates for specified ECs have been observed, with prospects of economic advantages and a better energy saving potential compared to state of the art UV/AOP implementations. The actual oxidizing effect of dissolved sodium hypochlorite (NaOCl) is based on the formation of hypochlorous acid (HOCl). The transformation of chlorine (Cl_2_) in an aqueous solution into hypochlorous acid and hydrochloric acid (HCl) is shown in Equation (1) [21]. The dissociation of hypochlorous acid into hypochlorite anions (ClO^−^) is pH dependent (Equation (2)) (pK_a_ = 7.5) [22]. ClO^−^ is a less effective oxidant, which is why at higher pH values the oxidation capability decreases [23]. In samples with pH values around 7, the predominant species is HOCl [23].

Cl_2_ + H_2_O → HOCl + Cl^−^ + H^+^ (1)

HOCl → ClO^−^ + H^+^ (2)

In the UV/chlorine process, inter alia, •OH and Cl• radicals are formed. Among others, the following reactions occur [24,25,26]:

HOCl + UV photons → •OH + Cl•(3)

ClO^−^ + UV photons → •O^−^ + Cl• (4)

•O^−^ + H_2_O → •OH + OH^−^ (5)

However, little research has been done in the study of UV/chlorine AOP treatment on wastewater at pilot scale. In this study, a continuous flow UV pilot plant was placed on the premises of a wastewater treatment plant. The goal was to examine the on-site feasibility of the UV/chlorine AOP applied to municipal WWTE.

## 2. Materials and Methods

### 2.1. Experimental Concept

The pilot plant, equipped with a medium pressure UV chamber with a maximum effective power of 1 kW, processed 1 m^3^/h in all experiments. The gas pressure inside the UV lamp has a significant influence on the spectral emittance of mercury. In comparison to low pressure UV lamps, which emit at one single wavelength (254 nm), medium pressure UV lamps emit at a broader spectrum (200–400 nm). When free chlorine (HOCl and ClO^−^) is dosed to wastewater, it can partially or fully react with wastewater components. The remaining active free Cl_2_ is mostly referred to as “free available chlorine” (FAC). In UV/chlorine experiments of this work, NaOCl solution was dosed to obtain the desired FAC concentrations in the UV chamber influent. In the case of an incomplete reaction of FAC in the UV chamber, “residual free chlorine” (RFC) in the UV chamber effluent could occur. This RFC was quenched according to Equation (6) [27] and Equation (7) [28] by means of an additional dosage of H_2_O_2_ downstream of the UV chamber.

HOCl + H_2_O_2_ → Cl^−^ + H_2_O + O_2_ + H^+^ (6)

Cl• + H_2_O_2_ → Cl^−^ + HO_2_• + H^+^ (7)

In parallel UV/H_2_O_2_ experiments, the oxidant dosed was H_2_O_2_ to allow for comparisons with the state of the art UV/H_2_O_2_ AOP. Two experiments were carried out, in which different experimental conditions were applied (no UV and no oxidant dosage; sole UV treatment; sole FAC or H_2_O_2_ treatment; and combinations out of UV, FAC and H_2_O_2_) as follows:

- Experiment 1: Variation of UV energy consumption (0.0, 0.4, 0.7, and 1.0 kWh/m^3^) at 0 and 3 mg/L oxidant concentrations (FAC or H_2_O_2_).
- Experiment 2: Variation of oxidant concentration (1–6 mg/L FAC or H_2_O_2_) at 0.4 kWh/m^3^ UV energy consumption.

Besides the removal of emerging contaminants, other impacts were examined. This was done with the aim to get a wide spectrum of the influence of the UV/chlorine and UV/H_2_O_2_ AOPs on WWTE using a continuous flow pilot plant. Cumulative effects on the WWTE were considered with the analysis of the bacterial count (in Supplementary Data), the total estrogenic activity, the formation of adsorbable organohalogens (AOX), combined Cl_2_ and chlorine oxyanions.

### 2.2. Chemicals and Reagents

NaOCl solution (14% active chlorine) was purchased from VWR International (Radnor, PA, USA) and H_2_O_2_ solution (35% technical grade) was received from Siemens Water Technologies (Günzburg, Germany). Sodium thiosulfate (Na_2_S_2_O_3_∙5H_2_O, ≥99%) was purchased from Carl Roth (Karlsruhe, Germany) and nitric acid solution (65%, p.a.) was purchased from Merck (Darmstadt, Germany). *N*,*N*-diethyl-*p*-phenylenediamine (DPD) was contained in powder pillows obtained by Hach (Berlin, Germany).

### 2.3. Wastewater Treatment Plant Effluent (WWTE) and Emerging Contaminants (ECs)

The state of the art municipal Treatment Plant for Education and Research (LFKW, Lehr- und Forschungsklärwerk) (primary clarifier, activated sludge treatment: denitrification/nitrification, P precipitation, secondary clarifier, micro sieves) lies on the premises of ISWA next to Büsnau, a district in Stuttgart, Germany. The average amount of treated wastewater in a year is about 900,000 m^3^ with a capacity of 30 L/s (9000 population equivalents). The raw wastewater of the LFKW is a mixture of domestic wastewater with a relatively high organic load and wastewater which is less concentrated from the university grounds. With the last treatment step of the LFKW, the water is filtered via micro sieves (15–20 µm pore size).

In Table 1, the initial parameter values c_0_ of the UV pilot plant influent for both AOP experiments are shown. The UV/H_2_O_2_ experiments were performed two months after the UV/chlorine experiments. Thus, both WWTEs differed slightly from each other. Nevertheless, apart from the temperature most of the parameters were very similar.

**Figure 1:**
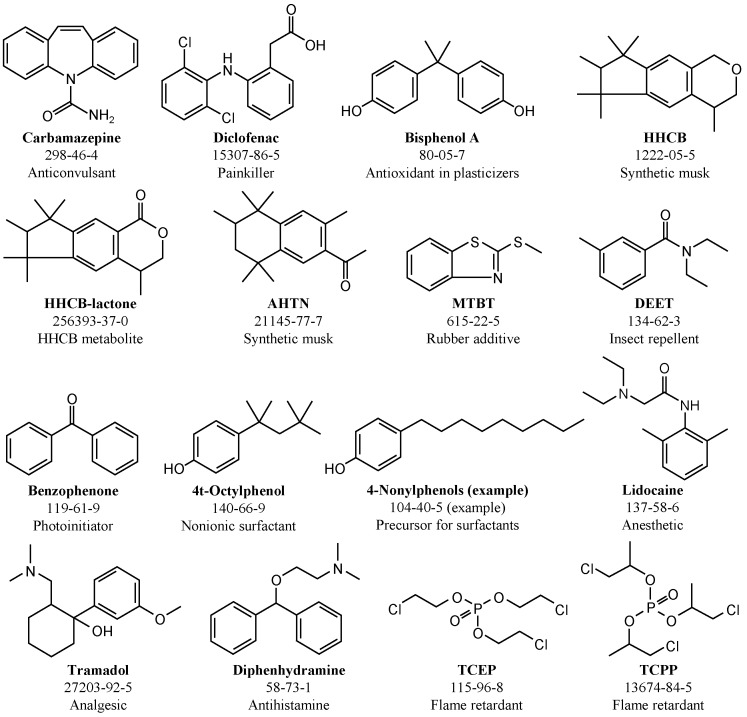
Overview of analyzed emerging contaminants with CAS numbers (based on [29]).

In Figure 1, an overview of the ECs analyzed in this study is given. ECs can be found in WWTE in various concentrations depending on the sampling time. In this study, the initial concentrations varied from 0.04 to 2.6 µg/L (Table 2). Most of the initial concentrations did not differ significantly between the experiments. Exceptions were the insect repellent DEET (0.04–1.99 µg/L) and the organophosphorous compound TCEP (0.35–1.76 µg/L).

### 2.4. UV Pilot Plant

**Figure 2:**
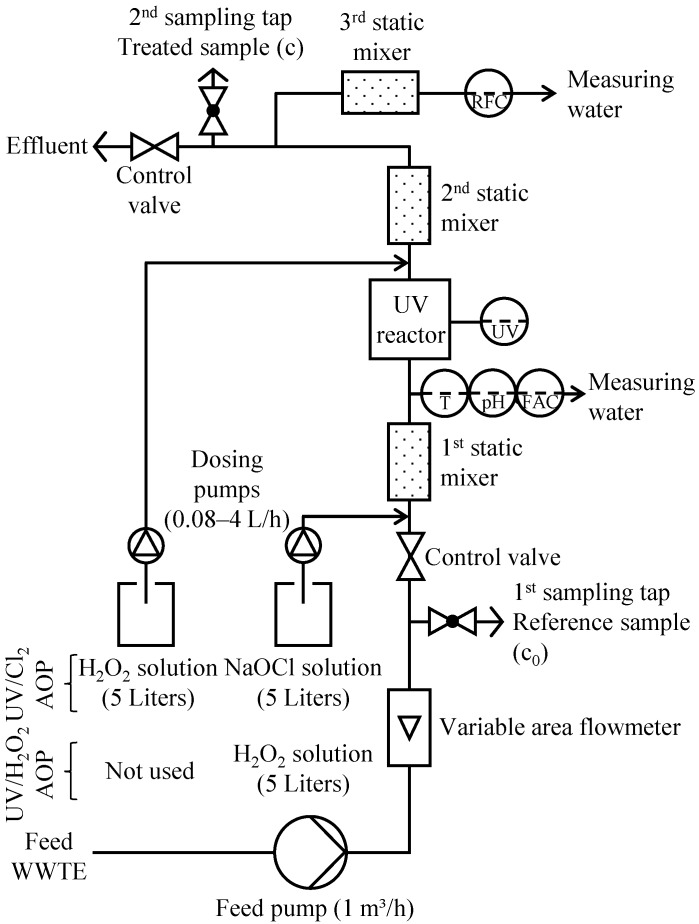
Technical scheme of the UV/chlorine AOP and UV/H_2_O_2_ AOP pilot plant used in the experiments.

The pilot plant (Figure 2) was placed in a hall where the micro sieves of the LFKW are situated. It was fed with the effluent of the micro sieves using an eccentric screw pump (Moineau pump) with the flow rate of 1 m^3^/h. The untreated reference sample (c_0_) could be collected from a tap behind the variable area flowmeter. In UV/chlorine AOP experiments, chlorine was dosed from a NaOCl stock solution (5–10 g/L free Cl_2_) with a peristaltic pump (0.08–4 L/h). In UV/H_2_O_2_ AOP experiments, this peristaltic pump was used for the dosage of H_2_O_2_ stock solution (5–10 g/L H_2_O_2_). The NaOCl (or H_2_O_2_) dosed water passed a static mixer to guarantee an extensive mixing through turbulence. The temperature, the pH (single junction, combination electrode sensor) and the FAC concentration (potentiostatic electrode amperometry sensor) of the UV chamber influent were determined by two membrane sensors (Wallace & Tiernan, Günzburg, Germany). At a flow rate of 1 m^3^/h, the contact time of chlorine until reaching the UV chamber was about 4.6–6.4 s. The brand-new immersion UV lamp (Wallace & Tiernan Barrier M35, type: WTL 1000, Siemens Water Technologies, Günzburg, Germany), protected by a quartz sleeve with a thickness of 1 mm and cut-off at 200 nm wavelength, was installed in a stainless steel chamber. The quartz sleeve could be cleaned by pushing an attached rubber ring back and forth. The irradiance could be controlled by a UV signal visualized on the cabinet determined by a 4–20 mA UV sensor (signal in W/m^2^). The approximate contact time in the UV chamber was 6–10 s. Downstream of the UV chamber, H_2_O_2_ could be dosed to the water to quench RFC in the pilot plant effluent and make it thus less harmful. In UV/H_2_O_2_ experiments, no H_2_O_2_ dosage was performed here. The focus of this study was on the technical feasibility of the UV/chlorine process by applying a continuous flow pilot plant. Therefore, the peristaltic pump for quenching agent dosage was mainly operated in automatic mode. This H_2_O_2_ dosage was automatically controlled by means of a chemical feed analyzer (for the RFC concentration) and process controller (MFC Analyzer/Controller) from Wallace & Tiernan (the RFC concentration of the pilot plant effluent was determined downstream of two further static mixers in a measuring cell with a potentiostatic electrode amperometry sensor). The FAC concentration could vary during an experiment while the H_2_O_2_ dosage was running and the experiments were limited in time. It was therefore not possible to determine the RFC concentration on a regular basis in case of missing H_2_O_2_ dosage. This aspect is therefore not addressed in this article. The contact time of the quenching agent from its dosage point to the effluent of the pilot plant was approximately 4.8–6.7 s. The treated sample (c) was collected from a second sampling tap. A control valve at the effluent was used to adjust the pressure in the system to enable a uniform distribution of water into all outgoing branches of the measuring cells of the pilot plant. At a flow rate of 1 m^3^/h, the approximate flow time from the pilot plant influent to the pilot plant effluent was 25–29 s.

### 2.5. Experimental Procedure

#### 2.5.1. Variation of UV Energy Consumption at 0 and 3 mg/L Oxidant Concentrations (Experiment 1)

The UV/chlorine AOP and the UV/H_2_O_2_ AOP were compared in this experiment. At first, the flow rate was adjusted to 1 m^3^/h. The oxidant concentration of 3 mg/L FAC or 3 mg/L H_2_O_2_ as well as the quenching agent dosage (not in UV/H_2_O_2_ AOP) was set. As soon as the pilot plant had reached a state of equilibrium (desired oxidant concentration achieved; no RFC measured in the pilot plant effluent), three reference samples (c_0_) and the first three treated samples (c) (3 mg/L oxidant, 0.0 kWh/m^3^) were taken from the sampling taps (separate bottles per analysis parameter). Then, the UV lamp was switched on and set to 0.4 kW. When the UV signal had stabilized, the next three treated samples were taken (3 mg/L oxidant, 0.4 kWh/m^3^, 90 ± 10 W/m^2^). Next, the UV lamp was set to 0.7 kW, and after 5 min, the next three treated samples were taken (3 mg/L oxidant, 0.7 kWh/m^3^, 140 ± 10 W/m^2^). This procedure was repeated with 1.0 kW (3 mg/L oxidant, 1.0 kWh/m^3^, 185 ± 15 W/m^2^). Then, the dosage of oxidant and quenching agent was switched off while the UV lamp was still running at 1.0 kW. After 10 min, the next three treated samples were collected (0 mg/L oxidant, 1.0 kWh/m^3^, 185 ± 15 W/m^2^). Subsequently, the UV lamp was set to 0.7 kW, and after 10 min, the next three treated samples were taken (0 mg/L oxidant, 0.7 kWh/m^3^, 140 ± 10 W/m^2^). This procedure was repeated with 0.4 kW (0 mg/L oxidant, 0.4 kWh/m^3^, 90 ± 10 W/m^2^). Finally, the UV lamp was switched off. After a further 10 min, three control samples (0 mg/L oxidant, 0.0 kWh/m^3^) could be taken. During all settings, only small pH drifts with a maximum pH of 7.6 were measured in the pilot plant effluent.

#### 2.5.2. Variation of Oxidant Concentration at 0.4 kWh/m^3^ UV Energy Consumption (Experiment 2)

In this experiment, the UV/chlorine AOP and the UV/H_2_O_2_ AOP were compared at 0.4 kWh/m^3^ UV energy consumption and varying oxidant concentrations (1–6 mg/L). At first, the flow rate was adjusted to 1 m^3^/h. Next, the UV lamp was switched on and set to 0.4 kW while the desired oxidant concentration (1 mg/L) and quenching agent dosage (not in UV/H_2_O_2_ AOP) was set. After at least 5 min, the pilot plant had reached a state of equilibrium (desired oxidant concentration achieved; no RFC measured in the pilot plant effluent; stable UV signal in the range of 90 ± 10 W/m^2^). Samples were then taken: For each analysis parameter, first, three sample bottles were filled with reference sample (c_0_) consecutively from a sampling tap. Subsequently, for each analysis parameter, three sample bottles were filled with treated sample (c) from a different sampling tap at the effluent of the pilot plant. Then, the next desired oxidant concentration of 2 mg/L was set while the UV lamp was still running. When the pilot plant had reached a state of equilibrium, the treated samples could be collected in the same way as described above. These steps were repeated for the oxidant concentrations of 3, 4, 5 and 6 mg/L. During all settings, only small pH drifts with a maximum pH of 7.6 were measured in the pilot plant effluent.

### 2.6. Analytical Methods

#### 2.6.1. Free Cl_2_, Combined Cl_2_, Total Cl_2_

Cl_2_, HOCl and OCl^−^ are referred to as free Cl_2_. Free Cl_2_ becomes combined Cl_2_ (CC, e.g., organic and inorganic chloramines) when it reacts with compounds in the water sample. Free Cl_2_ and combined Cl_2_ are summed up as total Cl_2_. In this study, a Hach DPD powder pillow method (photometer: Merck SQ 118) was used for the measurement of free Cl_2_ and total Cl_2_ equivalent concentrations and the calibration of the free Cl_2_ sensors on-site. In the following, there is also discussion of dosed Cl_2_ (Equations (8) and (9)). Not all halogenated products can be determined by the total Cl_2_ DPD method. Therefore, the dosed concentration of free Cl_2_ from the NaOCl stock solution is presented (c_dos_), which was calculated by means of Equation (10). c_sol_ is the free Cl_2_ concentration of the dosed NaOCl stock solution. Q_dos_ is the flow rate of the dosing pump, Q the flow rate of the pilot plant (1 m^3^/h). The term “Other Cl-containing reaction products” (OCRP) describes all substitution or oxidation/reduction products of the dosed Cl_2_ containing the element chlorine which cannot be detected as total Cl_2_ (e.g., chloride).

dosed Cl_2_ (c_dos_) = FAC/RFC + CC + OCRP (8)

dosed Cl_2_ (c_dos_) = total Cl_2_ + OCRP (9)

(10) $$c_{\mathrm{dos}}=\frac{c_{\mathrm{sol}}\times Q_{\mathrm{dos}}}{Q}$$

During all experiments, on-site free Cl_2_ and total Cl_2_ measurements were carried out almost at about every process setting when a certain state of equilibrium was reached.

#### 2.6.2. Chlorite (ClO_2_^−^), Chlorate (ClO_3_^−^), Perchlorate (ClO_4_^−^)

Chlorine oxyanions were determined only in the samples treated with 5 and 6 mg/L FAC at 0.4 kW UV power and their related reference sample in the 1st UV/chlorine AOP experiment using the standardized ISO 10304 method [30]. Each sample was filtered using C18 solid phase extraction cartridges and subsequent nylon filters with a pore size of 0.45 µm. The anions were detected by means of the Dionex ion chromatography system ICS-1000 (Waltham, MA, USA). An AS19a column (length: 25 cm, diameter: 2 mm) with a precolumn with anion self-regenerating suppressor was used. The gradient program was applied via a reagent-free controller. Each sample was measured twice. The limit of detection (LOD) for ClO_2_^−^ was 0.2 mg/L; for ClO_3_^−^, it was 0.06 mg/L; and, for ClO_4_^−^, it was 0.13 mg/L.

#### 2.6.3. Emerging Contaminants (ECs)

One-liter samples were quenched with 15 mg sodium thiosulfate (Na_2_S_2_O_3_). The determination of ECs was performed via gas chromatography directly coupled with a mass selective spectrometer (GC Hewlett Packard 5890N Series II, Hewlett Packard 5972 Series detector, column: Varian VF-Xms, length: 30 m, diameter: 0.25 mm, film thickness: 0.25 µm). After the addition of internal standards, the samples were liquid–liquid extracted (dichloromethane, 2 × 40 mL) and evaporated to 100 µL. Quantification was done using the isotope dilution method and external calibration. The limit of quantification (LOQ) was 1 ng/L.

#### 2.6.4. Total Estrogenic Activity (TEA)

For the determination of the total estrogenic activity, 1 L samples were collected without pretreating them prior to the analysis. The extracts obtained by solid-phase extraction were examined using an in vitro test system (E-screen assay) developed by Soto et al. [31] based on the instructions of Körner et al. [32] with modifications [33]. Thereby, the estrogenic activity reflects a sum parameter over all hormonal active compounds present in the samples expressed in concentration units of the reference compound 17β-estradiol. The LOQ of this method was 0.1 ng/L EEQ (17β-estradiol equivalent).

#### 2.6.5. Adsorbable Organohalogens (AOX)

All samples were filled into 300 mL BOD bottles and acidified with 3 drops of 35% nitric acid (HNO_3_) considering that the samples were free of headspace when the bottles were closed with glass stoppers. The determination of AOX concentrations was carried out using a standardized method [34]. In this method, the adsorbed and acidified sample is burned. Subsequently, the halogenide ions are determined via argentometry by means of microcoulometry (multi X 2000, Analytik Jena, Jena, Germany). The LOQ was 10 µg/L.

#### 2.6.6. Number of Measurements

The given values in diagrams or tables are mean values calculated from determinations of three equivalent samples taken consecutively. Error bars in diagrams and numbers after the “±” symbol in tables correspond to the calculated standard deviation.

## 3. Results and Discussion

### 3.1. Chlorine Species and Adsorbable Organohalogens (AOX)

**Figure 3:**
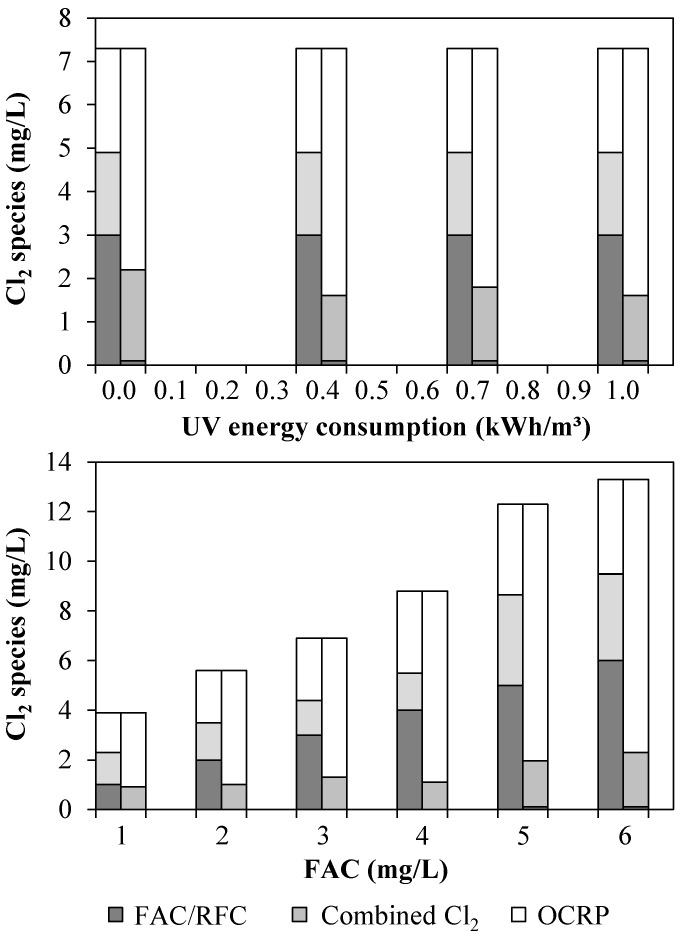
Chlorine species in UV chamber influent (1 m^3^/h WWTE) (left columns) and in effluent of the pilot plant after UV treatment and subsequent quenching with H_2_O_2_ (right columns): (**Top**) Experiment 1 (0–1 kW, 3 mg/L FAC in UV chamber influent); and (**Bottom**) Experiment 2 (0.4 kW, 1–6 mg/L FAC in UV chamber influent).

In Figure 3, for both UV/chlorine AOP experiments, the left columns depict the measured concentrations of FAC, combined Cl_2_ and OCRP in the UV chamber influent. The right columns show the concentrations of these chlorine species in the pilot plant effluent after quenching. In the upper diagram (Experiment 1), it can be seen that, during the entire UV/chlorine AOP experiment, a dosage of about 7 mg/L was required to obtain a concentration of 3 mg/L FAC in the UV chamber influent. RFC could be eliminated successfully. However, about 1.9 mg/L of added free Cl_2_ reacted to form compounds that could be measured as combined Cl_2_ via the DPD method. The removal extent of combined Cl_2_ was 10–20% with no significant effect of varying the UV performance (0.4, 0.7, and 1.0 kWh/m^3^). With the UV lamp switched off, no removal of combined Cl_2_ occurred. In Experiment 2 (lower diagram), to achieve a particular FAC concentration in WWTE, regardless of the desired FAC concentration, double to triple the amount of dosed Cl_2_ was needed. In this experiment, the removal extent of combined Cl_2_ varied between 10% and 50%, showing no linear correlation with the FAC concentration.

**Figure 4:**
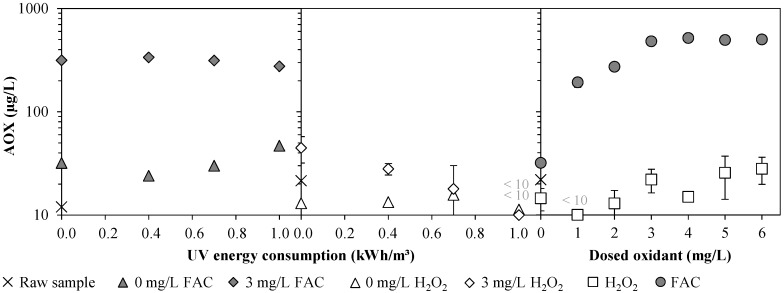
(**Left**, **Middle**) (Experiment 1) Influence of UV/chlorine AOP and UV/H_2_O_2_ AOP at 0.0, 0.4, 0.7, and 1.0 kWh/m^3^ UV energy consumption (1 m^3^/h, 0–1 kW) on AOX concentration in WWTE at oxidant concentrations of 0 and 3 mg/L; and (**Right**) (Experiment 2) influence of UV/chlorine AOP and UV/H_2_O_2_ AOP at 0.4 kWh/m^3^ UV energy consumption (1 m^3^/h, 0.4 kW) on AOX concentration in WWTE as a function of oxidant concentration.

Figure 4 sums up the measured concentrations of adsorbable organohalogens (AOX) in WWTE treated by the UV/chlorine AOP and the UV/H_2_O_2_ AOP. In all experiments, the AOX concentrations in the reference samples of the WWTE were very low, not exceeding 30 µg/L. In two control samples (sample of 2nd sampling tap, no oxidant dosage and no UV light), slightly higher AOX concentrations compared to the reference samples could be observed. However, the difference was insignificant since in all of these samples the AOX concentration was very low with <22 µg/L. In Experiment 1, both approaches with sole UV treatment (Figure 4, left and middle) led to AOX concentrations of <10–50 µg/L. However, considering the error susceptibility of the AOX determination method, the measured AOX concentrations were in such a small range that no significant AOX formation solely by UV treatment should be deduced. Furthermore, while the UV/H_2_O_2_ AOP had no significant effect on the AOX formation, already the sole dosage of FAC resulted in an AOX concentration of 314 ± 6 µg/L. Since only a slightly higher AOX concentration of up to 336 ± 6 µg/L was found with the UV/chlorine AOP at 0.4 kWh/m^3^ and even lower values of down to 276 ± 6 µg/L were found at 1.0 kWh/m^3^, it can be concluded that the formation of AOX is more due to chlorination and less to radical reaction. Much more, a higher UV power seemed to contribute to a reduction in AOX concentration. However, this reduction was very small. In Experiment 2 (right diagram), the UV/H_2_O_2_ AOP treatment did not increase the AOX concentration of the WWTE significantly as well (<30 µg/L AOX). In contrast, in WWTE samples treated with the UV/chlorine AOP, AOX could be measured up to 520 µg/L at FAC concentrations between 3 and 6 mg/L. With doses higher than 3 mg/L FAC, it seems as if a maximum AOX concentration was reached. This suggests that with 3 mg/L FAC most of the compounds in the WWTE that were available for chlorination were chlorinated. Furthermore, the two AOX concentrations at 0.4 kWh/m^3^ and 3 mg/L FAC from Experiment 1 (336 ± 6 µg/L) and Experiment 2 (480 ± 32 µg/L) were different. Since both experiments were performed on different days, the slightly different wastewater composition (different amounts of chlorinable compounds) may have contributed to the deviation in results. This deviation, however, is not critical since the found AOX concentrations are in a similar range. In conclusion, although the pilot plant design did not allow to take samples directly in front of the UV chamber to analyze the AOX formation between the Cl_2_ dosing point and UV chamber influent, there is considerable evidence that, despite the short contact time of 4.6–6.4 s, the AOX was formed before the UV chamber was reached, and this AOX formation was largely independent of the UV power.

The measured AOX concentrations were not high enough to fully explain the measured combined Cl_2_ concentrations in the UV chamber influent and pilot plant effluent. In addition, the very low ammonium concentration (<0.15 mg/L NH_4_^+^-N) in the WWTE does not justify the conclusion that the measured combined Cl_2_ consisted solely of inorganic chloramines. Furthermore, urea, a common precursor for inorganic chloramines, should not be found in WWTE due to its good removal in the wastewater treatment plant mainly based on hydrolysis [35]. The method for the determination of total Cl_2_ is based on the principle that chloramines are capable of oxidizing iodide ions, dosed parallel to the DPD, to iodine, which also forms a red dye in reaction with DPD. Thus, it is useful to consider further compounds having an oxidative effect as a possible cause for the detection of combined Cl_2_, i.e., also non-Cl-containing compounds [36]. To comprehend some of these oxidative by-products detected as combined Cl_2_, the concentrations of the conjugated bases of oxyacids of chlorine (CBO) were determined in samples treated with 5 and 6 mg/L FAC at 0.4 kWh/m^3^ (Experiment 2) (<0.2 mg/L ClO_2_^−^, 1.03 ± 0.02 mg/L ClO_3_^−^, <0.26 mg/L ClO_4_^−^ at 5 mg/L FAC and <0.2 mg/L ClO_2_^−^, 1.11 ± 0.02 mg/L ClO_3_^−^, and <0.26 mg/L ClO_4_^−^ at 6 mg/L FAC). In these samples, the measured concentrations of combined Cl_2_ in the UV chamber influent were 3.7 ± 0.6 mg/L (at 5 mg/L FAC) and 3.5 ± 0.7 mg/L (at 6 mg/L FAC). In the corresponding samples of the UV pilot plant effluent, the combined Cl_2_ concentrations were 1.9 ± 0.1 mg/L (at 5 mg/L FAC) and 2.2 ± 0.1 mg/L (at 6 mg/L FAC). From the experiment, it cannot be said whether chlorate was formed by chlorination, photolysis or radicals since this compound was only analyzed in the pilot plant effluent at parallel UV and chlorine dosage and not in the UV chamber influent. According to the literature, however, it can be assumed that UV light contributes significantly to its formation [24]. This indicates chlorate was surely hardly present in the UV chamber influent, where despite of that relatively high combined Cl_2_ concentrations were present. Furthermore, it is obvious that also H_2_O_2_, which was dosed to the UV chamber effluent to quench RFC, contributed its part to increase the total Cl_2_ concentration value. Approximately 9.1–9.2 mg/L H_2_O_2_ (about 270 μmol/L) were added at FAC dosages of 5 and 6 mg/L (70–85 µmol/L) at 0.4 kWh/m^3^. According to Equation (6), this was 3–4 times the concentration that would be stoichiometrically required to quench 5–6 mg/L free Cl_2_. Thus, surplus H_2_O_2_ must have been present in the pilot plant effluent, which must have contributed to a slightly falsified value of total Cl_2_. In an experiment (Figure S1), this falsification was quantified to be 0.0388 mg total Cl_2_/mg H_2_O_2_, so that at 9.1–9.2 mg/L H_2_O_2_ only a maximum interference of about 0.35 mg/L CC could be present. In the pilot plant effluent samples, which were treated with 5 and 6 mg/L FAC at 0.4 kWh/m^3^, 1.9–2.2 mg/L combined Cl_2_ was measured. With less than 20%, the falsification was therefore not large enough to fully explain the found concentration of combined Cl_2_ in the pilot plant effluent and especially not in the UV chamber influent, where no H_2_O_2_ was present in the UV/chlorine AOP.

The relatively high concentration of combined Cl_2_ in both the UV chamber influent and the pilot plant effluent can be attributed to a wide variety of other degradation products. Such disinfection by-products (DBP) resulting from the chlorination process are described in great detail in the literature. Trihalomethane (THM) formation is more pronounced in WWTEs with very low ammonium concentrations than in those with high ammonium concentrations [37]. The study of this work is based on WWTE with a very low NH_4_^+^-N concentration (<0.15 mg/L), so it can be assumed that THMs were prominently represented. It is also known that many dissolved organic nitrogen compounds (DON) in WWTE are essential precursors for N-DBPs [38,39]. In the effluent of the WWTP examined here, the annual average DON concentration was 1.6 mg/L (monthly average values varied between 0.9 and 2.4 mg/L N) and was thus in a range similar to the concentration of combined Cl_2_ found. Extensive research by Pehlivanoglu-Mantas and Sedlak [40] showed that up to 10–20% of the DON concentration in WWTE can be attributed to amino acids and thus constitute a considerable precursor pool for the formation of N-DBPs such as dihaloacetonitriles [37,38,39]. This is relevant because amino acids have a relatively high reactivity with HOCl (k_app_ > 1 × 10^4^ M^−1^ s^−1^) [23]. Other important known DBPs that may have been formed during the chlorination of WWTE are trichloronitromethanes, haloketones and chloral hydrates [17].

### 3.2. Emerging Contaminants

**Figure 5:**
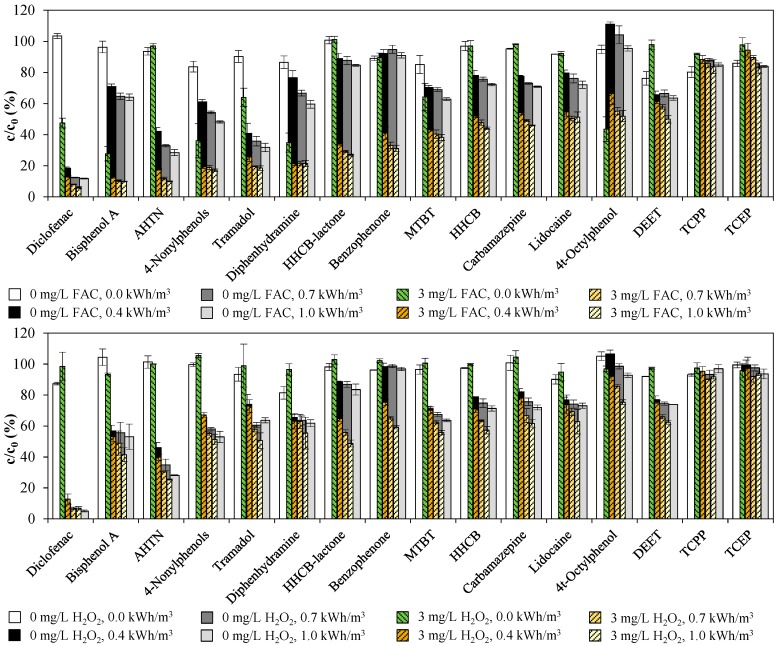
Removal of ECs found in WWTE by means of: UV/chlorine AOP (**top**); and UV/H_2_O_2_ AOP (**bottom**), at 0 and 3 mg/L oxidant concentrations depending on UV energy consumption at 1 m^3^/h (Experiment 1).

Figure 5 sums up the relative residual concentrations of ECs found in WWTE after the treatment with the UV/chlorine AOP (Figure 5, top) and the UV/H_2_O_2_ AOP (Figure 5, bottom) with different UV performances (0.0, 0.4, 0.7, and 1.0 kWh/m^3^). Except for DEET, no compound could be eliminated more than 20% when no oxidant dosage was applied and the UV lamp was off. This indicates that only small reductions of the initial concentrations could be caused by adsorption processes in the static mixers. Some compounds could already be degraded by treating WWTE only with 3 mg/L FAC, such as diclofenac, bisphenol A, MTBT, 4t-octylphenol, 4-nonylphenols, tramadol and diphenhydramine. These are chemicals with electron-rich moieties (phenols, anilines, amines) that are preferably attacked by the selective chlorine molecule [41]. In the case of sole H_2_O_2_ dosage, no such effect was observed. Sole UV treatment reduced the concentration of many compounds to a certain degree. Exceptions were 4t-octylphenol, benzophenone, TCEP and TCPP. Bisphenol A, AHTN, 4-nonylphenols, tramadol and especially diclofenac were very susceptible to sole UV radiation. In comparison to sole UV treatment, the additional dosage of FAC paralleled with UV radiation was highly effective for almost all compounds except DEET. The UV/H_2_O_2_ AOP was only partly effective for ECs like HHCB, HHCB-lactone, benzophenone and 4t-octylphenol (only with high energy consumption), but not effective for any other ECs analyzed. Furthermore, the removal extents resulting from 3 mg/L oxidant and 0.4 kWh/m^3^ were not increased more than 15 percentage points by increasing the energy consumption up to 1 kWh/m^3^.

**Figure 6:**
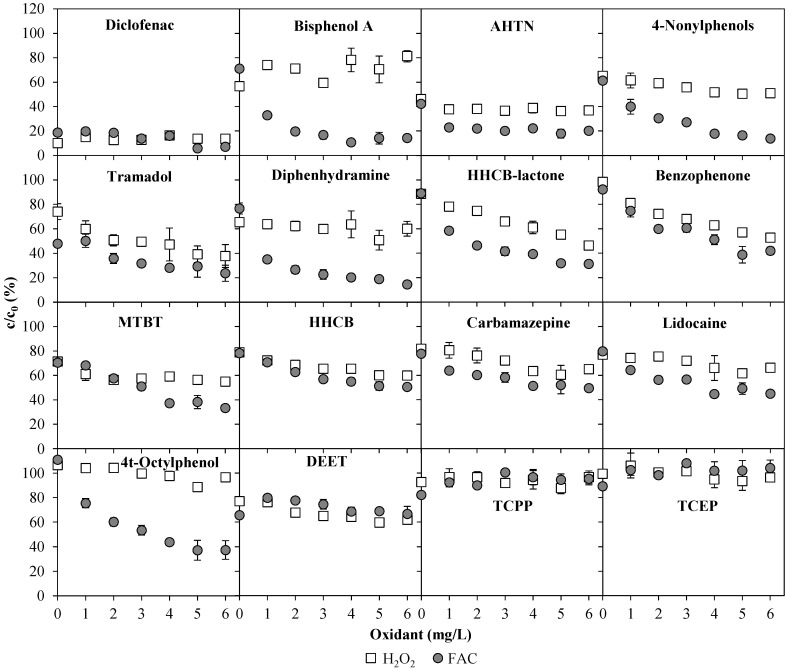
Removal of emerging contaminants found in WWTE by means of UV/chlorine AOP and UV/H_2_O_2_ AOP at 0.4 kWh/m^3^ UV energy consumption (1 m^3^/h, 0.4 kW) as a function of oxidant concentration (Experiment 2). At 0 mg/L oxidant: sole UV treatment.

In Figure 6, the removal of the same ECs found in WWTE due to the UV/chlorine AOP and UV/H_2_O_2_ AOP treatment at 0.4 kWh/m^3^ energy consumption (1 m^3^/h, 0.4 kW) is shown as a function of the oxidant concentration from 0 to 6 mg/L. The organophosphorous compounds TCEP and TCPP could not be eliminated with both AOPs. Both latter ECs are nevertheless designed to be resistant against oxidation [10]. Most of the partially eliminated compounds were degraded more effectively by the UV/chlorine AOP (exceptions were diclofenac and DEET). While 4t-octylphenol was not affected by the UV/H_2_O_2_ AOP, the UV/chlorine AOP treatment resulted in a 65% removal of that compound. The xenoestrogens bisphenol A and 4-nonylphenols could be removed by means of the UV/chlorine AOP by up to almost 90%. For most of the ECs, significantly higher removal extents could be achieved with higher oxidant concentrations. However, there were also compounds that did not seem to be affected by the variation of the oxidant concentration. Such compounds such as AHTN and diclofenac showed the same reaction for both the UV/chlorine AOP and the UV/H_2_O_2_ AOP. Additionally, these compounds also underwent degradation due to sole UV exposure (0 mg/L oxidant). The results obtained in Experiment 2 for 3 mg/L FAC or 3 mg/L H_2_O_2_ at 0.4 kWh/m^3^ UV energy consumption did not differ more than 10% from the results of Experiment 1 in Figure 5. Both experiments were carried out on different days with slightly different wastewater compositions, which indicates a good reliability of the results.

In several publications, the very good EC elimination potential of the UV/chlorine AOP observed in this study could be seen as well (see Table S1 in Supplementary Materials summarizing results from other studies investigating the removal of ECs by the UV/chlorine AOP) [16,17,18,19,20]. However, none of these studies examined the UV/chlorine AOP on WWTE with such a high variation of analyzed ECs without spiking them and also applying the AOP at pilot scale. In this study, the initial concentration of diclofenac could be reduced up to 90% with both AOPs (most of the elimination can be attributed to photolysis). This pharmaceutical proved to be very susceptible to sole UV exposure, which can be ascribed to photoactive chromophores contained in the molecule [42]. As Sichel et al. [16] and Zhou et al. [43] had already observed, carbamazepine spiked in tap water or pure water could not significantly be eliminated with sole Cl_2_ treatment (no UV exposure) even within a reaction time of 60 min. This poor degradation could be seen in this study as well, where contact times lower than 1 min were present. Here, carbamazepine could be eliminated by a maximum of approximately 50% with the application of the UV/chlorine AOP at the highest tested FAC concentration of 6 mg/L. A complete elimination of carbamazepine by the UV/chlorine AOP was only detected by Wang et al. [19]. However, their experiments were carried out with pure water and longer contact times.

The compounds diclofenac (DCF), bisphenol A (BPA) and 4-nonylphenols (4-NPh) were already eliminated at 50–70% with a dosage of 3 mg/L FAC. This happened at a contact time of about 9–16 s. The rate constants found in the literature for chlorination of these compounds at pH 7 were all determined at initial concentrations of ECs that were more than a hundred times higher than in the WWTE of this study (k_app,DCF_ = 3.5 M^−1^ s^−1^ [44], k_app,BPA_ = 62 M^−1^ s^−1^ [45], k_app,4-NPh_ = 12.6 M^−1^ s^−1^ [46]). The resulting half-lives are >4 min and cannot be compared with the results gained in this study describing very small EC concentrations. Some investigations are available that examine the kinetics of the removal of ECs by the UV/chlorine AOP, with distinctions into chlorination, photolysis and radical reaction [18,19,43]. These investigations were usually carried out with much weaker UV lamps and simpler matrices than WWTE. Wang et al. [19], e.g., found a rate constant of k_obs_ = 0.78 min^−1^ for the degradation of 2 mg/L carbamazepine (8.5 µM) by 280 µM Cl_2_ and 1.48 mW/cm^2^ (41 W) in pure water matrix. At a contact time of 6–10 s, as in the UV chamber of this study, they found less than 15% degradation of carbamazepine, whereas in this study even at 42 µM FAC an elimination of 46% occurred. A direct transferability of the rate constants available in the literature is therefore not possible here either.

At a UV energy consumption of 0.4 kWh/m^3^ and in a range of oxidant concentrations from 1 to 6 mg/L, the UV/chlorine AOP had a much better EC removal yield than the UV/H_2_O_2_ AOP for most of the analyzed compounds. This especially occurred with xenoestrogens like bisphenol A and 4-nonylphenols, which could be degraded very effectively. The more pronounced degradation yield by the UV/chlorine AOP compared to the UV/H_2_O_2_ AOP even at lower molar concentrations of FAC compared to H_2_O_2_ was also observed by Sichel et al. [16], Yang et al. [17] and Xiang et al. [18] (Table S1 in Supplementary Materials). Some possible reasons for this could be: The more efficient •OH radical yield due to different quantum yields at a wavelength of 254 nm and lower scavenger rates in the UV/chlorine AOP compared to the UV/H_2_O_2_ AOP [16,47]. Furthermore, other studies applying the UV/H_2_O_2_ AOP to real wastewater also showed that far higher concentrations of H_2_O_2_ were required for successful degradation yields than the 6 mg/L H_2_O_2_ used in this study [48,49].

### 3.3. Total Estrogenic Activity in the UV/Chlorine AOP Experiments

**Figure 7:**
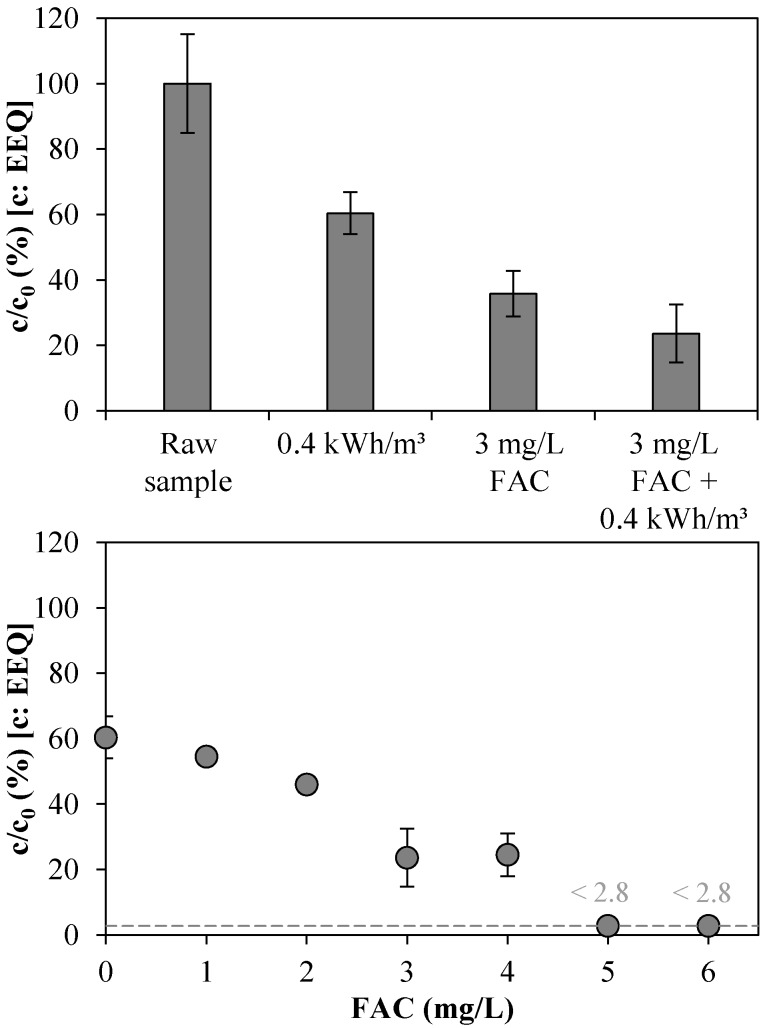
Effect of UV/chlorine AOP on total estrogenic activity of WWTE: measured in 17β-estradiol equivalents (EEQ) at different process settings at 1 m^3^/h (**Top**) (Experiment 1); and as a function of FAC concentration in UV chamber influent at 0.4 kWh/m^3^ energy consumption (1 m^3^/h, 0.4 kW) (**Bottom**) (Experiment 2). Dashed line: LOQ of 0.1 ng/L EEQ (here 2.8%).

The total estrogenic activity (TEA) was analyzed in samples from UV/chlorine AOP experiments (Figure 7). With 65% EEQ removal, the sole dosage of 3 mg/L FAC had a stronger elimination effect than sole UV radiation at 0.4 kWh/m^3^ (40% EEQ removal). This shows that xenoestrogens lose their estrogenic activity when they become chlorinated [50]. The UV/chlorine AOP could reduce the TEA of WWTE from 40% to at least 97% in the FAC concentration range of 0–6 mg/L at 0.4 kWh/m^3^ (initial concentrations of 3.92 ± 0.31 and 1.77 ± 0.25 ng/L EEQ, respectively).

It is striking that those compounds that are known for their estrogenic activity were among those compounds that were removed best (60–90%) with the UV/chlorine AOP. These are in particular the endocrine disrupting compounds (EDCs) bisphenol A (BPA), 4-nonylphenols (4-NPh), and 4t-octylphenol (4t-OctPh) [51]. Similar to most estrogenic compounds, these EDCs bear phenolic hydroxyl groups. Lee et al. [52] suspected that the phenolic ring is oxidized by chlorination (it is likely that phenolic rings are preferably oxidized by chlorine [52]) in such a way that the TEA decreases. Wu et al. [50] also suspected that the EDCs are converted by chlorination into less estrogenic by-products leading to a decrease in TEA. Furthermore, Li et al. [20] had shown that the UV/chlorine AOP was the most efficient method compared to sole Cl_2_ dosage and sole UV exposure in reducing estrogenic activity, even in the presence of NH_3_ and wastewater matrix (Table S1 in Supplementary Materials). The reaction was very fast: the majority of the reaction was completed within less than 1 min. Similar contact times were observed for the pilot plant discussed here. Thus, in combination with the study of Li et al. [20], this work showed that even with complex matrixes such as WWTE and applied with a continuous mode pilot plant, the UV/chlorine AOP is an effective method for reducing the TEA.

Rosenfeldt and Linden [53], Rosenfeldt et al. [54] and Cédat et al. [48] showed with the EDCs 17β-estradiol, 17α-ethinylestradiol, bisphenol A and estrone that also the UV/H_2_O_2_ AOP, with sufficient dosage of H_2_O_2_, significantly decreases the estrogenic activity compared to sole UV treatment. However, Rosenfeldt et al. [54] also found that the reduction of estrogenic activity in wastewater matrix is significantly weaker compared to pure water matrix. In this study, the dosage of 3 mg/L H_2_O_2_ was obviously too low even at 1 kWh/m^3^ UV exposure to significantly degrade the aforementioned EDCs (BPA, 4-NPh, 4t-OctPh). If one takes into account the statement of Lee et al. [52] that the elimination of estrogenic chemicals correlates with the elimination of TEA, it can thus be assumed that the UV/chlorine AOP reduces the TEA stronger than the UV/H_2_O_2_ AOP.

## 4. Conclusions

Effluent of a municipal wastewater treatment plant was treated with the UV/chlorine AOP on a technical scale on-site using a medium pressure UV lamp with an adjustable performance of up to 1 kW. In parallel experiments with the same pilot plant, the UV/H_2_O_2_ AOP was applied for comparison. The UV/chlorine AOP proved to be a highly effective method regarding the removal of bacteria (see Figure S2) and the removal of the estrogenic activity and thus endocrine disrupting compounds from WWTE. Compared to the UV/H_2_O_2_ AOP, most of the analyzed emerging contaminants were removed more efficiently with the UV/chlorine AOP. By-products in the form of AOX (most likely mainly by chlorination) and chlorate (most likely mainly by photolysis) occurred. Metabolites are of great concern regarding methods based on the oxidation of ECs [55], therefore, treatment of WWTE solely by the UV/chlorine AOP must be considered critically. Since AOX have a high tendency towards adsorption on activated carbon, an activated carbon treatment subsequent to the UV/chlorine AOP is recommended. Furthermore, with such a combination, compounds such as TCEP and TCPP can be eliminated as well [56], despite their high stability against UV/chlorine oxidation. Chlorine is known to react quickly with ammonium ions [57]. Especially in cold seasons, high ammonium concentrations can occur in WWTE. However, formed chloramines also have an oxidizing potential and can be transformed to radicals under UV exposure [47,58]. Thus, the effect of high ammonium concentrations on the effectiveness of the UV/chlorine AOP pilot plant still needs more research.

## Reference

## Figures and Tables

**Table 1:** Initial parameter values c_0_ measured in WWTE reference samples collected in both UV/chlorine AOP and UV/H_2_O_2_ AOP experiments (n. m.: not measured; COD: chemical oxygen demand; DOC: dissolved organic carbon; EEQ: 17β-estradiol equivalent; AOX: adsorbable organohalogens; * only one determination).

Parameter	Variation of UV Energy Consumption between 0 and 1 kWh/m^3^ (Experiment 1)		Variation of Oxidant Concentration at 0.4 kWh/m^3^ (Experiment 2)		
	0 and 3 mg/L FAC	0 and 3 mg/L H_2_O_2_	1–4 mg/L FAC	5–6 mg/L FAC	1–6 mg/L H_2_O_2_
Temperature (°C)	14.9	18.9	14.6	14.8	19.5
pH	7.0	7.0	7.0	7.0	7.0
COD (mg/L)	17.8 ± 1.3	20.4 ± 1.8	23.6 ± 0.3	23.2 ± 0.1	21.3 ± 0.8
DOC (mg/L)	5.8 ± 0.9	5.9 ± 0.3	6.0 ± 1.1	6.9 ± 0.2	5.5 ± 0.1
NH_4_^+^-N (mg/L)	<0.15	<0.11	<0.1	<0.1	<0.1
EEQ (ng/L)	1.83 ± 0.28	n. m.	3.92 ± 0.31	1.77 ± 0.25	n. m.
AOX (µg/L)	12 *	25 ± 1	21 ± 5	25 ± 5	22 ± 8
ClO_2_^−^ (mg/L)	n. m.	n. m.	n. m.	<0.20	n. m.
ClO_3_^−^ (mg/L)	n. m.	n. m.	n. m.	<0.06	n. m.
ClO_4_^−^ (mg/L)	n. m.	n. m.	n. m.	<0.13	n. m.

**Table 2:** Initial EC concentrations c_0_ measured in WWTE reference samples collected in both UV/chlorine AOP and UV/H_2_O_2_ AOP experiments (three samples with single determination).

Emerging Contaminant (µg/L)	Variation of UV Energy Consumption between 0 and 1 kWh/m^3^ (Experiment 1)		Variation of Oxidant Concentration at 0.4 kWh/m^3^ (Experiment 2)		
	0 and 3 mg/L FAC	0 and 3 mg/L H_2_O_2_	1–4 mg/L FAC	5–6 mg/L FAC	1–6 mg/L H_2_O_2_
Carbamazepine	0.47 ± 0.00	0.48 ± 0.02	0.75 ± 0.02	0.83 ± 0.02	0.43 ± 0.02
Diclofenac	1.16 ± 0.02	2.28 ± 0.09	2.15 ± 0.08	2.55 ± 0.18	1.83 ± 0.14
Bisphenol A	0.77 ± 0.00	0.61 ± 0.04	0.85 ± 0.02	0.57 ± 0.30	0.53 ± 0.12
HHCB	1.20 ± 0.01	1.14 ± 0.02	1.24 ± 0.03	1.19 ± 0.06	1.12 ± 0.02
HHCB-lactone	1.33 ± 0.02	1.21 ± 0.03	1.61 ± 0.05	1.56 ± 0.09	1.20 ± 0.08
AHTN	0.16 ± 0.00	0.14 ± 0.00	0.18 ± 0.01	0.18 ± 0.01	0.14 ± 0.01
MTBT	0.24 ± 0.00	0.29 ± 0.01	0.24 ± 0.01	0.22 ± 0.03	0.28 ± 0.01
DEET	0.08 ± 0.00	1.99 ± 0.01	0.05 ± 0.00	0.04 ± 0.01	0.28 ± 0.02
Benzophenone	0.14 ± 0.00	0.20 ± 0.00	0.12 ± 0.00	0.13 ± 0.02	0.21 ± 0.01
4t-Octylphenol	0.04 ± 0.00	0.04 ± 0.00	0.04 ± 0.00	0.05 ± 0.01	0.03 ± 0.00
4-Nonylphenols	1.93 ± 0.03	1.67 ± 0.06	1.65 ± 0.13	1.55 ± 0.24	2.01 ± 0.12
Lidocaine	0.12 ± 0.01	0.22 ± 0.00	0.27 ± 0.00	0.23 ± 0.01	0.17 ± 0.01
Tramadol	0.11 ± 0.01	0.17 ± 0.01	0.16 ± 0.01	0.09 ± 0.02	0.21 ± 0.02
Diphenhydramine	0.25 ± 0.01	0.25 ± 0.01	0.22 ± 0.01	0.27 ± 0.02	0.24 ± 0.01
TCEP	1.06 ± 0.02	0.69 ± 0.02	1.76 ± 0.03	0.35 ± 0.04	1.27 ± 0.16
TCPP	0.91 ± 0.05	1.56 ± 0.05	1.42 ± 0.02	1.66 ± 0.22	1.17 ± 0.09

## Supplementary Materials

Supplementary Data regarding influence of H_2_O_2_ on total Cl_2_ analysis, results of UV/chlorine AOP in literature and elimination of bacterial count are available online at http://www.mdpi.com/1660-4601/15/5/935/s1. Table S1: Comparison of the results of different studies regarding the removal of important ECs (in %) by the UV/chlorine AOP and UV/H_2_O_2_ AOP (all studies except for this study and the study of Sichel et al. [16] were conducting batch experiments). Table S2: Initial bacterial count measured in WWTE reference samples collected in both UV/chlorine AOP and UV/H_2_O_2_ AOP experiments (CFU: colony forming units). Figure S1: Detected total Cl_2_ concentrations in samples with different H_2_O_2_ concentrations without chlorine compounds. Figure S2: (Left, Middle) (Experiment 1) Influence of UV/chlorine AOP and UV/H_2_O_2_ AOP at 0.0, 0.4, 0.7, and 1.0 kWh/m^3^ UV energy consumption (1 m^3^/h, 0–1 kW) on bacterial count in WWTE at oxidant concentrations of 0 and 3 mg/L; and (Right) (Experiment 2) influence of UV/chlorine AOP and UV/H_2_O_2_ AOP at 0.4 kWh/m^3^ UV energy consumption (1 m^3^/h, 0.4 kW) on bacterial count in WWTE as a function of oxidant concentration.
