# Supplementary material for: Removal of Emerging Contaminants and Estrogenic Activity from Wastewater Treatment Plant Effluent with UV/Chlorine and UV/H2O2 Advanced Oxidation Treatment at Pilot Scale
Source: Int J Environ Res Public Health. 2018 May 7;15(5):935. doi: 10.3390/ijerph15050935 (PMC5981974; doi:10.3390/ijerph15050935)
Supplement: Supplementary file 1 [file ijerph-15-00935-s001.pdf]

International Journal of
Environmental Research and Public Health

MDPI

Supplementary Material to the Article

Removal of Emerging Contaminants and Estrogenic Activity from Wastewater Treatment Plant Effluent with UV/Chlorine and UV/H_2O_2 Advanced Oxidation Treatment at Pilot Scale

Eduard Rott *, Bertram Kuch, Claudia Lange, Philipp Richter, Amélie Kugele and Ralf Minke

Institute for Sanitary Engineering, Water Quality and Solid Waste Management, University of Stuttgart, Bandtäle 2, 70569 Stuttgart, Germany

* Correspondence: eduard.rott@iswa.uni-stuttgart.de; Tel.: +49-711-685-60497

A. Influence of H_2O_2 on Total Cl_2 Analysis

A.1. Experimental Procedure

One tablet of $H_2O_2{\cdot}CO(NH_2)_2$ (Merck KGaA, Darmstadt, Germany) was dissolved in 1 L pure water and from this stock solution (0.3381 g/L H_2O_2 & 0.5970 g/L $CO(NH_2)_2$) eight different dilutions with H_2O_2 concentrations between 2.5 and 50 mg/L were prepared. The urea concentration in these dilutions varied accordingly between 4.4 and 88.3 mg/L. In addition, 1 L of stock solution was prepared only with 0.5970 g/L $CO(NH_2)_2$ (Merck KGaA, Darmstadt, Germany), which was also divided into several 100 mL dilutions with a maximum urea concentration of 88.3 mg/L. Each dilution was analyzed with the DPD method for total Cl_2 and sporadically some dilutions were analyzed with the DPD method for free Cl_2.

A.2. Results

Figure S1 clearly shows that H_2O_2 in a sample leads to the finding of total Cl_2 even though no chlorine compounds are present in the sample. Free Cl_2 was not detected in these samples (not shown in the figure). In the samples with up to 88.3 mg/L urea (without H_2O_2), no total Cl_2 and no free Cl_2 were detected (not shown in the figure). The finding of total Cl_2 in the solutions with H_2O_2 and urea can therefore be attributed solely to H_2O_2. The measured total Cl_2 correlates with the H_2O_2 concentration in the form of 0.0388 mg total Cl_2/mg H_2O_2.

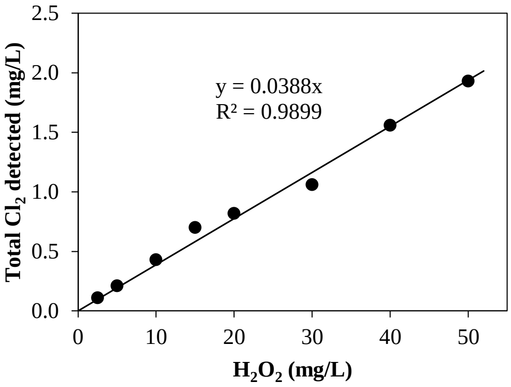

Figure S1. Detected total Cl_2 concentrations in samples with different H_2O_2 concentrations without chlorine compounds.

B. UV/Chlorine AOP in the Literature

Table S1. Comparison of the results of different studies regarding the removal of important ECs (in %) by the UV/chlorine AOP and UV/H_2O_2 AOP (all studies except for this study and the study of Sichel et al. [16] were conducting batch experiments).

Reference	Process	Oxidant	UV radiation	Matrix	Time	CBZ	DCF	IBP	E2
This study	Cl_2	42 μM FAC*	-	WWTE	1 m^3/h	2	53		
	H_2O_2	88 μM H_2O_2	-	WWTE	1 m^3/h	0	0		
	UV	-	400 W, 0.4 kWh/$m^{3\#}$	WWTE	1 m^3/h	22	82		
	UV	-	1000 W, 1 kWh/$m^{3\#}$	WWTE	1 m^3/h	29	88		
	UV/Cl_2	42 μM FAC*	400 W, 0.4 kWh/$m^{3\#}$	WWTE	1 m^3/h	46	87		
	UV/Cl_2	42 μM FAC*	1000 W, 1 kWh/$m^{3\#}$	WWTE	1 m^3/h	54	94		
	UV/H_2O_2	88 μM H_2O_2	400 W, 0.4 kWh/$m^{3\#}$	WWTE	1 m^3/h	22	87		
	UV/H_2O_2	88 μM H_2O_2	1000 W, 1 kWh/$m^{3\#}$	WWTE	1 m^3/h	38	93		
Sichel et al. (2011) [16]	Cl_2	85 μM Cl_2	-	TW	15 min	4	32		
	UV/H_2O_2	147 μM H_2O_2	80 W, 0.32 kWh/m^3	TW	250 L/h	59	100		
	UV/Cl_2	14 μM Cl_2	80 W, 0.32 kWh/m^3	TW	250 L/h	48	100		
	UV/H_2O_2	147 μM H_2O_2	40 W, 0.16 kWh/m^3	TW	250 L/h	31	89		
	UV/Cl_2	85 μM Cl_2	40 W, 0.16 kWh/m^3	TW	250 L/h	90	100		
	UV/Cl_2	85 μM Cl_2	40 W, 0.16 kWh/m^3	TW+DOC[a]	250 L/h	82	100		
Wang et al. (2016) [19]	UV	-	41 W, 1.48 mW/cm^2	PW	5 min	0			
	Cl_2	280 μM Cl_2	-	PW	5 min	6			
	UV/Cl_2	280 μM Cl_2	41 W, 1.48 mW/cm^2	PW	5 min	100			
	UV/Cl_2	280 μM Cl_2	41 W, 1.48 mW/cm^2	PW+tBuOH[b]	5 min	12			
Zhou et al. (2016) [43]	UV	-	75 W, 1.14 mW/cm^2	PW	60 min	0			
	Cl_2	500 μM Cl_2	-	PW	60 min	42			
	UV/Cl_2	500 μM Cl_2	75 W, 1.14 mW/cm^2	PW	60 min	84			
	UV/Cl_2	500 μM Cl_2	75 W, 1.14 mW/cm^2	PW+DOC[c]	60 min	39			
Yang et al. (2016) [17]	UV	-	10 W	WWTE	1.5 min	1–8			
	Cl_2	71 μM Cl_2	-	WWTE	1.5 min	0–10			
	UV/Cl_2	71 μM Cl_2	10 W	WWTE	1.5 min	30–60			
	UV/H_2O_2	147 μM H_2O_2	10 W	WWTE	1.5 min	10–25			
Xiang et al. (2016) [18]	UV/H_2O_2	100 μM H_2O_2	10 W, 1.05 mW/cm^2	PW (pH 6)	20 min			69	
	UV/Cl_2	100 μM Cl_2	10 W, 1.05 mW/cm^2	PW (pH 6)	20 min			99	
	UV/Cl_2	100 μM Cl_2	10 W, 1.05 mW/cm^2	PW (pH 7)	20 min			70	
	UV/Cl_2	100 μM Cl_2	10 W, 1.05 mW/cm^2	TW (pH 7)	20 min			60	
Li et al. (2016) [20]	UV	-	2.1 mW/cm^2	PW	9 min				10
	Cl_2	141 μM Cl_2	-	PW	9 min				99
	UV/Cl_2	141 μM Cl_2	2.1 mW/cm^2	PW	9 min				99
	UV	-	2.1 mW/cm^2	WWTE	9 min				13
	Cl_2	141 μM Cl_2	-	WWTE	9 min				45
	UV/Cl_2	141 μM Cl_2	2.1 mW/cm^2	WWTE	9 min				90

FAC: Free available chlorine, TW: Tap water, PW: Pure water, WWTE: Wastewater treatment plant effluent, CBZ: Carbamazepine, DCF: Diclofenac, IBP: Ibuprofen, E2: 17β-Estradiol, * 103 μM free Cl_2 dosed, # at 0.4 kWh/m^3: 9 ± 1 mW/cm^2 (6–10 s); and at 1 kWh/m^3: 18.5 ± 1.5 mW/cm^2 (6–10 s), [a] 46 mg/L DOC (100 mg/L citric acid and 40 mg/L urea), [b] 741 mg/L tBuOH, [c] 10 mg/L DOC (diluted river sample).

C. Bacterial Count

C.1. Analysis of Bacterial Count

Before they were filled with sample, 100 mL bottles were heated at 105 °C for at least 8 h in order to destroy bacteria which may affect the measurement. In a sterile environment, 100 μL of the sample were pipetted onto an agar culture medium. After 5 days of incubation at 37 °C, the colony forming units (CFU) were counted. The limit of detection was 10 CFU/mL. In this study, nutrient broth (NB) or lysogeny broth (LB) medium was used.

C.2. Results of Bacterial Count

Table S2 shows the initial CFU measured in the reference samples for both experiments.

Table S2. Initial bacterial count measured in WWTE reference samples collected in both UV/chlorine AOP and UV/H_2O_2 AOP experiments (CFU: colony forming units).

	**Variation of UV Energy Consumption between 0 and 1 kWh/m³ (Experiment 1)**		**Variation of Oxidant Concentration at 0.4 kWh/m³ (Experiment 2)**		
Parameter	0 and 3 mg/L FAC	0 and 3 mg/L H_2O_2	1–4 mg/L FAC	5–6 mg/L FAC	1–6 mg/L H_2O_2
Bact. count (CFU/mL)	1913 ± 64	2317 ± 234	1723 ± 196	1227 ± 215	1973 ± 6

In the right diagram of Figure S2, the effect of the UV/chlorine AOP and the UV/H_2O_2 AOP at a constant energy consumption (0.4 kWh/m³) on the bacterial count in WWTE can be seen. For both AOPs, two different mediums for the cultivation of bacteria were used: nutrient broth medium (NB) in the UV/H_2O_2 AOP experiment and lysogeny broth medium (LB) in the UV/chlorine AOP experiment. Since both mediums have a similar composition, no great effects on the results are to be expected. Both experiments confirmed that the UV/chlorine AOP has a strong disinfecting effect. While the dosage of 1 mg/L FAC at an energy consumption of 0.4 kWh/m³ had already reduced the bacterial count to less than 10 CFU/mL, at a concentration of 6 mg/L H_2O_2 at 0.4 kWh/m³ UV energy consumption more than 100 CFU/mL were counted. Hence, applying the UV/chlorine AOP the bacterial count could be reduced by up to 3 $\log_{10}$ stages.

Figure S2 compares the influence of the UV/chlorine AOP (left) and the UV/H_2O_2 AOP (middle) on the bacterial count as a function of the UV energy consumption (0.0, 0.4, 0.7, 1.0 kWh/m³). While both the initial concentrations of colony forming units (CFU) in the reference samples were in the same range, the results obtained from UV exposure itself (triangles in the left and middle diagrams) did not correspond to each other. In the UV/H_2O_2 AOP experiment, about ten times the concentration of CFU was counted. Since there were two months between both experiments, it is possible that during the UV/H_2O_2 AOP experiment more UV resistant bacteria were present in the WWTE. Despite this incongruity, the conducted analysis, however, confirmed once more the good disinfecting effect of the UV/chlorine AOP (<10 CFU/mL). Even the sole presence of 3 mg/L FAC in WWTE, without being activated by UV radiation, led to a reduction of the bacterial count by almost 2 $\log_{10}$ stages. In the UV/H_2O_2 AOP experiments, a bacterial count elimination of only 1 $\log_{10}$ stage could be observed. The sole dosage of H_2O_2 did not have any effect.

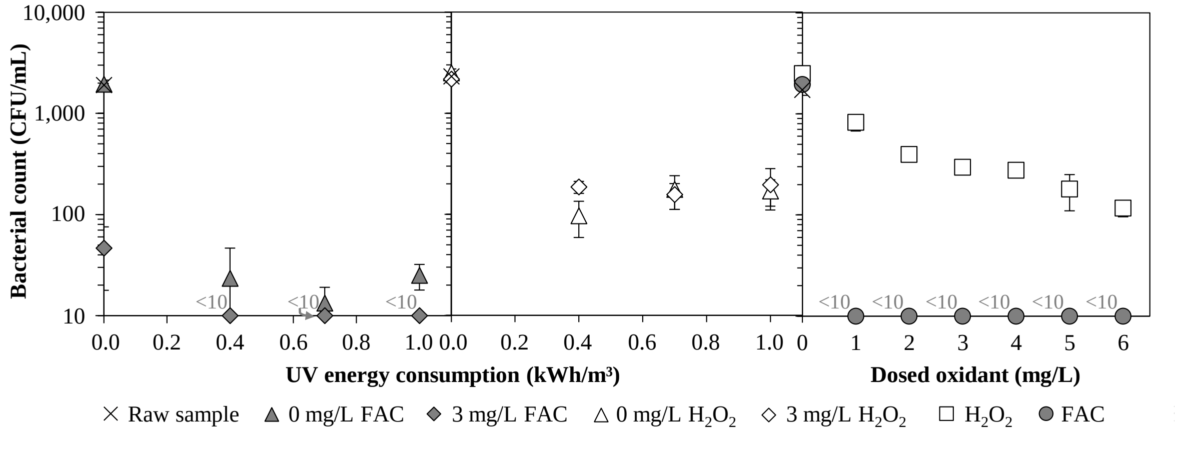

Figure S2. (**Left, Middle**) (Experiment 1) Influence of UV/chlorine AOP and UV/H_2O_2 AOP at 0.0, 0.4, 0.7, and 1.0 kWh/m³ UV energy consumption (1 m³/h, 0–1 kW) on bacterial count in WWTE at oxidant concentrations of 0 and 3 mg/L; and (**Right**) (Experiment 2) influence of UV/chlorine AOP and UV/H_2O_2 AOP at 0.4 kWh/m³ UV energy consumption (1 m³/h, 0.4 kW) on bacterial count in WWTE as a function of oxidant concentration.
